# An evolutionary case for functional gene body methylation in plants and animals

**DOI:** 10.1186/s13059-017-1230-2

**Published:** 2017-05-09

**Authors:** Daniel Zilberman

**Affiliations:** 1 John Innes Centre, Colney Lane, Norwich, NR4 7UH UK; 2 University of California, 211 Koshland Hall, Berkeley, CA 94720 USA

## Abstract

Methylation in the bodies of active genes is common in animals and vascular plants. Evolutionary patterns indicate homeostatic functions for this type of methylation.

Cytosine methylation is a covalent modification of DNA that is shared by plants, animals, and other eukaryotes [1]. The most frequently methylated sequences in plant genomes are symmetrical CG dinucleotides, and this methylation is maintained across cell divisions by the MET1 family of methyltransferases. Plants also have abundant methylation of cytosines in other (non-CG) sequence contexts, which is catalyzed by the chromomethylases (CMT2 and CMT3) and by the DRM enzymes that are guided by small RNA molecules via the RNA-directed DNA methylation (RdDM) pathway [2, 3].

Methylation in all contexts is located within transposable elements, which are nearly ubiquitously methylated in land plant genomes [1–3]. Methylation prevents transposon expression and transposition and is, therefore, essential for plant genome integrity and transcriptional homeostasis [2, 3]. DNA methylation of transposons that are close to or within genes can affect gene expression, in most cases causing silencing [2, 4]. Modulation of this type of methylation can regulate genes during development. For example, selective methylation removal in specialized sex cells activates some genes and silences others, a process that is essential for successful reproduction [4].

## Gene body methylation

In addition to transposons, DNA methylation frequently occurs in active plant genes [2, 3, 5]. Gene body methylation (GbM) has been most extensively explored in flowering plants, in which thousands of genes typically carry GbM in the CG context, with very low levels of accompanying non-CG methylation [2, 3, 5]. GbM is preferentially located in the exons of long and moderately expressed genes and away from the 5′ and 3′ gene ends [2, 3, 5, 6]. Perhaps the most interesting correlation is between GbM and gene responsiveness, a measure of gene expression variability in different cell types or environmental conditions. GbM is most frequent in constitutively expressed (i.e., housekeeping) genes, and least frequent in the genes with the most variable expression [2, 5]. Consistently, the amino acid sequences of methylated genes tend to evolve more slowly than those of unmethylated genes [2, 5, 6]. Recent analyses indicate that similar genes tend to be methylated in other vascular plants, such as ferns, although the associated levels of non-CG methylation are much higher [7]. These results suggest that GbM is a coherent and conserved phenomenon that encompasses at least 400 million years of land plant evolution.

## The debate about GbM functionality

The function of GbM has remained mysterious. Loss of GbM through mutation of *MET1* does not cause major alterations of steady-state mRNA levels in *Arabidopsis thaliana* [3, 5], and natural GbM variation in *Arabidopsis* populations does not correlate with gene expression [8]. Two flowering plant species lack GbM without apparent ill effects [9].

The inability to detect the functional consequences of GbM has prompted hypotheses that GbM has no function and arises as an inconsequential byproduct of spurious interactions between transposon methylation pathways, such as the chromomethylases or RdDM, and genes [3, 5, 9]. The main argument in favor of functionless GbM is that GbM is dispensable—genetically, but more importantly evolutionarily. However, loss and turnover are nearly ubiquitous evolutionary forces [10]. Snakes have lost legs, humans lack biosynthetic enzymes for several amino acids, and fruit flies have lost telomerase. DNA methylation itself has been lost in many eukaryotic lineages [1]. This does not mean that these features are not essential in the species that possess them.

One reason to be wary of drawing functional inferences from evolutionary loss is that biological features are replete with trade-offs. For example, silencing of invasive transposons by DNA methylation damages gene expression [2]. Functional pathways can be lost when the costs of the side effects closely match or outweigh the benefits. GbM almost certainly has major negative consequences because methylation increases the rate of C-to-T transition mutations [11]. As a result, the human genome has only a quarter of the expected CG sites [11]. Genic methylation increases the rates of deleterious human mutations, including those associated with cancer [11, 12], indicating an evolutionary cost. GbM mutagenizes plant genes as well: grass genes have long been known to belong to two categories, CG-rich and CG-poor, but the effect remained unexplained until the discovery that CG-poor genes exhibit GbM and CG-rich ones do not [6]. Without a countervailing selective benefit, why would GbM be specifically maintained in the exons of genes that are under strong selection against changes to encoded amino acids [6]?

One might argue that plants do not have a choice. DNA methylation is needed to silence transposons, and features of methylation pathways, such as the preferences of RdDM or the chromomethylases, may selectively target constitutively expressed genes. Features of these genes, for example, the higher CG content of exons, might in turn cause methylation to be preferentially maintained in exons. The increased mutational load associated with GbM would then be added to gene silencing as a cost of inhibiting transposition through DNA methylation. However, plants can modify methylation patterns via demethylating enzymes that counteract the gene-silencing effects of transposon methylation [2, 3]. *Arabidopsis* also possesses a protein that prevents the accumulation of high levels of non-CG methylation in the genes that exhibit CG GbM [2, 3]. Plants are clearly able to evolve mechanisms that remove deleterious methylation, including from gene bodies.

The notion of GbM as a tolerated side effect of transposon silencing becomes even less plausible if GbM in animal genomes is considered. Plants and animals are ancient groups that diverged over a billion years ago [1]. CG methylation is maintained in animal genomes by the same methyltransferase family as in plants, but animals lack chromomethylases and RdDM [1]. Despite these differences, animal GbM is strikingly similar to that of plants: methylation is preferentially found in the exons of modestly, constitutively expressed and evolutionarily conserved housekeeping genes [1, 13, 14]. GbM occurs in species that span roughly 900 million years of animal evolution, from cnidarians to chordates [1]. In some lineages, the most studied of which are the Hymenoptera (ants, bees, and wasps), methylation is very rare outside of genes [1, 14]. In these species, GbM cannot be a byproduct of functional methylation elsewhere. At least in the Hymenoptera, GbM must have a function that outweighs its mutational cost.

## Function of GbM

The above discussion should not be taken to mean that no functions have been ascribed to GbM. The clearest plant case of GbM functionality is in rice, where gene silencing is strongly associated with selective removal of GbM in female sex cells [4]. A similar, but much weaker, correlation has been observed in *Arabidopsis* [4]. Nonetheless, genes apparently silenced by GbM removal represent a small fraction of all methylated genes and GbM patterns at most genes probably remain constant across plant development [2, 4]. The constitutive expression and housekeeping functions of genes that are typically affected by GbM also suggest that the main function of GbM is not to modulate expression during development or in response to the environment. The function of GbM is most likely homeostatic.

Several homeostatic GbM functions have been proposed [2, 5]. One suggestion is that GbM may stabilize gene expression by preventing aberrant transcription from internal cryptic promoters. Another possibility is that GbM enhances splicing efficiency, as suggested by the preferential methylation of exons. GbM reduces the accumulation of histone variant H2A.Z, which is associated with highly responsive genes even in species without DNA methylation, suggesting that GbM may reduce expression variability by excluding H2A.Z. The above hypotheses have yet to be thoroughly tested. Cryptic transcripts are rapidly degraded and are not easily detected in RNA-seq data [15]. Mis-spliced transcripts with premature stop codons are also very unstable [15]. The stabilization of gene expression through H2A.Z exclusion is not expected to alter steady-state mRNA levels except on very short time scales, and thus would not be detected in data that averages transcription over many cells. Some or all of the proposed hypotheses may turn out to be wrong, but it is premature to conclude that any of them have been disproven [5] until they are tested with techniques that measure transcription rather than mRNA levels and are able to analyze small numbers of cells.

It is formally possible that GbM is maintained in some animal species because it has a function, but that methylation is located in similar genes of other animals, and of plants, as an unavoidable consequence of functionality elsewhere. It is possible that GbM has a function in animals, but not in plants despite the strong similarities. It is also possible that non-functional GbM has been nearly ubiquitous in vascular plant species over the last 400 million years despite littering the exons of some of the most essential and highly conserved genes with mutations. None of these possibilities appear very likely. Occam’s razor suggests that methylation has been maintained in constitutively expressed genes of plants and animals over hundreds of millions of years because methylation has a function in these genes. We should figure out what this function is.

## Abbreviations

GbM: Gene body methylation
RdDM: RNA-directed DNA methylation
